# High expression of SOX9 is a diagnostic and prognostic indicator of glioma

**DOI:** 10.3389/fonc.2025.1531937

**Published:** 2025-07-07

**Authors:** Libo Xu, Zhenhao Wang, Mao Li, Qingsong Li

**Affiliations:** ^1^ Department of Neurosurgery, The Second Affiliated Hospital of Harbin Medical University, Harbin, China; ^2^ College of Biological Sciences, University of California, Davis, Davis, United States

**Keywords:** GBM, SOX9, immune cell infiltration, prognostic mode, targeted therapies

## Abstract

**Background:**

Glioblastoma (GBM) originates from neuroepithelial tissue and is one of the most common intracranial malignant tumors in adults, with high recurrence rate and poor prognosis. In recent years, SOX9 has been reported to play an important role in many diseases and cancers, and is a promising target, but it has been rarely reported in GBM.

**Methods:**

RNA sequencing data of GBM were obtained from the Cancer Genome Atlas (TCGA) database and the Genotype-Tissue Expression (GTEx) database for analysis of SOX9 expression and differentially expressed genes (DEGs). Moreover, functional enrichment analysis of GBM-related DEGs was performed by GO/KEGG, GSEA, and protein-protein interaction (PPI) network. Additionally, the clinical significance of SOX9 in GBM was assessed by Kaplan-Meier Cox regression and prognostic model. What’s more, we analyzed SOX9-related immune cell infiltration and expression of immune checkpoints in GBM. The incorporated studies were analyzed using the R package.

**Results:**

SOX9 was highly expressed in a range of malignant tumor tissues, including GBM. Surprisingly, high SOX9 expression was remarkably associated with better prognosis in the lymphoid invasion subgroups in a sample of 478 cases (P < 0.05). Totally, 126 differentially significant genes (DSGs) were identified between high- and low- expression group, of which 29 genes were upregulated and 97 genes were downregulated. Furthermore, high expression of SOX9 was an independent prognostic factor for IDH (isocitrate dehydrogenase)-mutant in Cox regression analysis. Screening was performed by LASSO coefficients to select non-zero variables that satisfied the coefficients of lambda. min, and four genes were screened out. OR4K2 and IDH status were prognostic factors associated with THCA in multifactorial COX regression analysis. SOX9, OR4K2 and IDH status were included in the nomogram prognostic model. Correlation analysis indicated SOX9 expression was correlated with immune cell infiltration and expression of immune checkpoints in GBM.

**Conclusion:**

SOX9 was identified as a diagnostic and prognostic biomarker in glioblastoma, particularly in IDH-mutant cases. Its expression was closely correlated with immune infiltration and checkpoint expression, indicating its involvement in the immunosuppressive tumor microenvironment. SOX9-based gene signatures further supported a robust nomogram model, underscoring its potential as a therapeutic and prognostic target in GBM.

## Introduction

Gliomas are the most common primary central nervous system (CNS) malignancies, accounting for about 40% of intracranial tumors (1). The characteristics of gliomas include a high incidence, invasiveness, a high rate of recurrence, an extremely short overall survival (OS) time, and a high 5-year mortality rate (2). The current standard of treatment includes maximal surgical resection and combined radio chemotherapy (3, 4). However, there has been no significant improvement in prognosis during these decades, which necessitates additional investigation (5, 6). In recent years, research at the molecular level has revealed that the pathogenesis of gliomas is driven by abnormal pathological processes, such as dysregulation of the cell cycle, signaling pathways, and other factors (7). Therefore, identifying new biomarkers may help to better understand the molecular basis of GBM, which could potentially play a crucial role in GBM diagnosis, prognosis, prediction of treatment responses, and the development of targeted therapies.

The SOX (SRY-related HMG-box) gene family is a group of nuclear transcription factors related to embryonic development. It is named after its homology with the SRY (sex-determining region of the Y chromosome) gene located on the male Y chromosome. Members of this family contain transcription factors with a highly conserved HMG (high-mobility group box) domain structure. This domain encodes a DNA-binding domain consisting of 79 amino acids, which can recognize specific DNA sequences in the genome and play a role in DNA binding and transcriptional regulation, among other functions (8). The SOX family plays a crucial role in embryonic development in various tissues and organs and serves as biomarkers for different types of tissue cells (9). Additionally, it constitutes an important group of stem cell transcription factors, with about 20 members capable of binding DNA through the HMG domain. Some of these members have been associated with tumorigenesis and metastasis (10, 11). The transcription factor SOX9 (SRY-related HMG-box 9) belongs to the SOX protein family, which includes SOX8, SOX9, SOX10, and SOXE (12). Fan et al. (13) have demonstrated that the loss of SOX9 in tumor cells can significantly reduce tumor cell metastasis. Zhou et al. (14) have found that SOX9 is upregulated in various types of cancers and significantly correlates with tumor grading and poorer overall survival rates in human lung adenocarcinoma (LUAD) patients. In addition, the research has also found that SOX9 suppresses the tumor microenvironment in lung adenocarcinoma and is mutually exclusive with various tumor immune checkpoints (15, 16). However, the relevant molecular mechanisms remain unclear in glioblastoma.

Therefore, this study aims to determine the correlation between SOX9 expression and the prognosis, immune infiltration, and immune checkpoint relevance in GBM. Firstly, we obtained RNA-seq data for GBM from TCGA and GTEx to analyze the expression of SOX9. In addition, functional enrichment analysis of SOX9 was conducted using GO, KEGG, and GSEA. We also analyzed immune cell infiltration and immune checkpoint analysis. Kaplan-Meier and COX regression analyses, as well as nomogram prediction model analysis, were employed to assess the clinical significance of SOX9 in GBM. Through these methods, we will identify significantly altered genes and pathways that may play a crucial role in the pathogenesis of GBM in association with SOX9.

## Materials and methods

### Tissue-specific expression of SOX9 and expression of SOX9 in GBM

To retrieve the transcriptomic expression levels of SOX9 in GBM, we utilized the Human Protein Atlas (HPA) (https://www.proteinatlas.org/) database. Additionally, to validate protein-level expression, we performed western blotting using GBM tumor tissues and adjacent normal brain tissues collected from clinical samples. The pan-cancer RNA-seq data were obtained from *TCGA* (https://portal.gdc.cancer.gov/) and *GTEx* (https://gtexportal.org/). HTSeq-FPKM and HTSeq-Count data of the GBM samples were acquired from the *TCGA* (https://portal.gdc.cancer.gov/repository) for further analysis. The study fully complies with the guidelines provided by TCGA and GTEx.

### Expression and enrichment analysis of SOX9 correlated genes

*LinkedOmics* (http://www.linkedomics.org/) (17) was used to assess and draw a heatmap of the top 35 positively/negatively related genes with SOX9. Related genes with the adjusted P-value <0.05 were applied for functional enrichment analysis. We used *Metascape* (https://metascape.org) (18) to visualize the enriched biological process (BP), cellular composition (CC), molecular function (MF) and KEGG pathway terms of the SOX family and its co-expressed genes.

### Differentially expressed gene analysis

The DESeq2 R package was adopted to compare expression data of low- and high-expression of COMMD7 (cut-off value of 50%) in AML samples (HTseq-Count) to identify DEGs (19). R package ggplot2 (3.3.6) was used to visualize volcano plots of the result.

### PPI network

The PPI network of DEGs was predicted using the Search Tool for the Retrieval of Interacting Genes (STRING) database (20). The interaction score threshold of 0.4 was set as the cut-off criterion. The PPI network was mapped using Cytoscape (version 3.7.1) (21), and the most significant modules in the PPI network were identified using MCODE (version 1.6.1) (22). Selection criteria were as follows: MCODE scores >5, degree cut-off = 2, node score cut- off = 0.2, Max depth = 100, and k-score = 2.

### Functional enrichment analysis

DEGs with the threshold for | log fold change (logFC)| >2 and adjusted P-value (adj P-value) <0.05 were applied for functional enrichment analysis. Gene Ontology (GO) functional analysis comprising cellular component (CC), molecular function (MF), and biological process (BP), as well as Kyoto Encyclopedia of Genes and Genomes (KEGG) pathway analysis, were implemented using the ClusteProfiler package in R (23).

### Gene set enrichment analysis

R package ClusteProfiler (3.14.3) was used for GSEA to elucidate the functional and pathway differences between the high- and low-expression groups of SOX9 (23). The gene set was permutated 1,000 times for each analysis. Adjusted P-value < 0.05 and FDR q-value < 0.25 were considered to be statistically significant.

### Correlation analyses for SOX9 expression and clinical features of GBM patients

SOX9 expression was compared between tumors and normal tissues by receiver operating characteristic (ROC) analysis to test the predictive value of SOX9 for GBM diagnosis. Clinicopathological characteristics were compared for high- and low- SOX9 expression groups using the Wilcoxon rank sum test (continuous variables) or Spearman chi-square test (rank variables). The correlation between GBM expression and clinicopathological characteristics was evaluated by logistic analysis. Kaplan–Meier (K-M) analysis, univariate, and multivariate Cox regression analysis were employed for prognosis analysis.

### Prognostic model generation and prediction

In order to individualize the prediction of overall survival (OS) in THCA patients, a nomogram was generated using the RMS R package (version 5.1-3), which included genes screened by LASSO coefficient filtering, prominent clinical characteristics and calibration plots. The calibration curves were evaluated graphically by mapping the nomogram-predicted probabilities against the observed rates, and the 45°line represented the best predictive values. Concordance index (C-index) was used to determine the discrimination of the nomogram, and the bootstrap approach was used to calculate 1000 resamples. In addition, C-index and receiver operating characteristic (ROC) were used to analyze and compare the predictive accuracy of the nomogram and separate prognostic factors. All statistical tests were double-tailed with 0.05 as the statistical significance level.

### Immune cell infiltration analysis and immune checkpoints expression analysis

The ssGSEA package and ESTIMATE package in the GSVA package [version 1.34.0] were used for immuno-infiltration correlation analysis of SOX9. The statistical significance of the difference was evaluated by Spearman’s test. In addition, Wilcoxon rank sum test was used to analyze the correlation between SOX9 expression and immune checkpoints expression in GBM. The correlation between SOX9 expression and immune checkpoints expression was evaluated by Spearman chi-square test.

### Drug sensitivity analysis

We used the GSCALite database (http://bioinfo.life.hust.edu.cn/web/GSCALite/) (24) and TISDIB database (http://cis.hku.hk/TISIDB/index.php) to analyze the correlation between SOX9 expression and sensitivity to current chemotherapeutic or targeted drugs for GBM.

### Western blot analysis

Tumor and brain tissues were lysed by 1% SDS lysis buffer and boiled at 95°C for 10 min, then centrifuged at 12,000 rpm for 10 min at room temperature. The protein concentration was determined by a BCA protein assay reagent kit (Beyotime Institute of Biotechnology, Jiangsu, China). The protein (35 µg) was separated by SDS-PAGE gels and transferred onto PVDF membranes (Merck, Darmstadt, Germany). The membranes were blocked for 2h by 5% fat free milk in TBST at room temperature, followed by incubation overnight at 4°C with the primary antibodies for anti-SOX9 (ABclonal, Wu Han, China; Cat# A19710) and anti-GAPDH (Cell Signaling Technology, Danvers, MA, USA; Cat# 2118). The membranes were washed with TBST and incubated for 1h with the corresponding HRP-conjugated second antibodies. Bands were visualized with ECL Reagents (Beyotime Institute of Biotechnology, Jiangsu, China; P0018S). Each Western blot experiment was independently repeated four times (N = 4) using patient-derived GBM and adjacent normal brain tissues.

### Statistical analysis

In this study, R (4.2.1) and corresponding R packages were utilized for statistical analysis. In all tests, P value < 0.05 was considered statistically significant.

## Results

### SOX9 expression in pan-cancers and GBM

**Figure 1:**
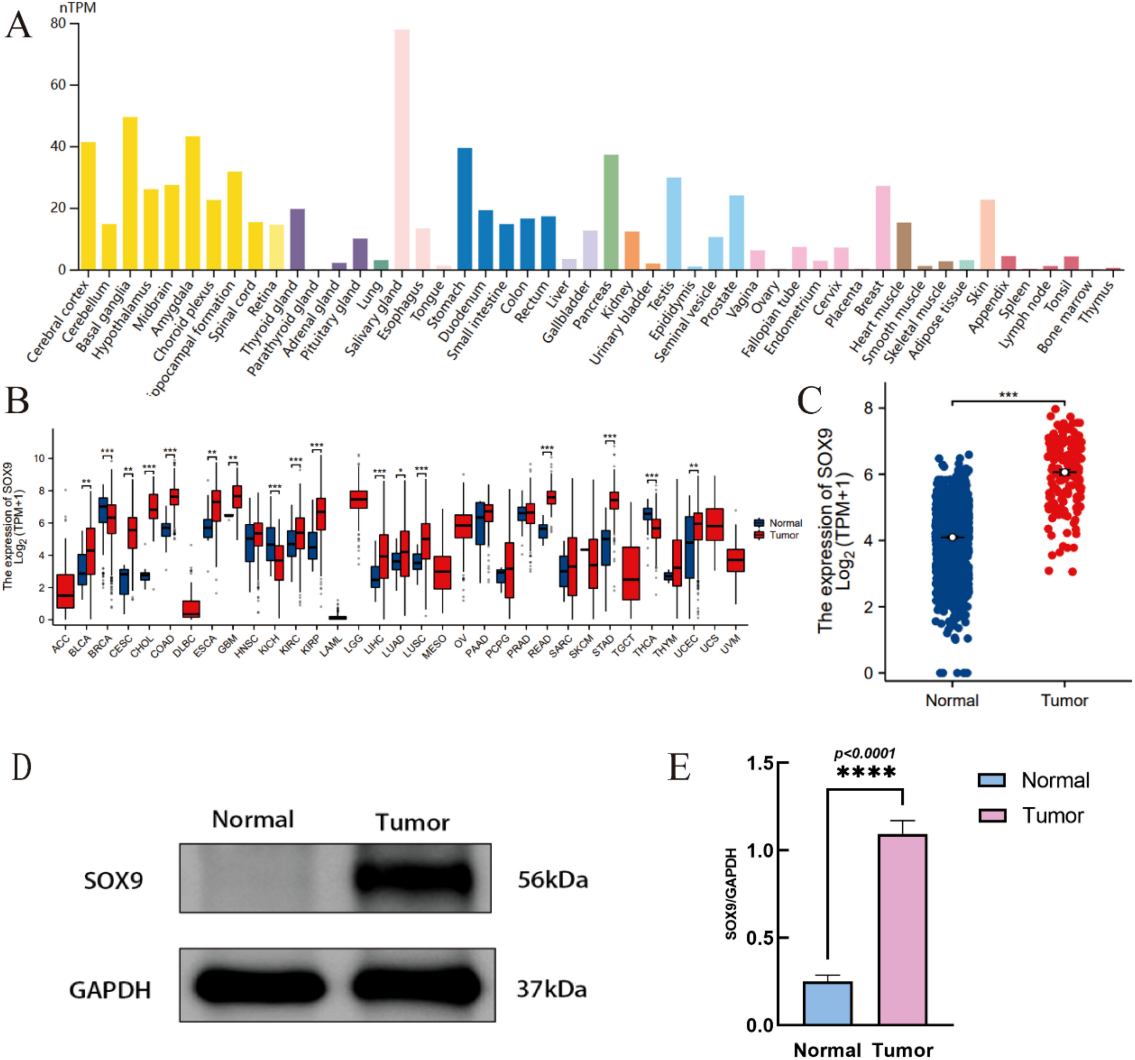
Expression of SOX9 in tissue and GBM. **(A)** tissue-specific expression Of SOX9; **(B)** expression of SOX9 in pan-cancer; **(C)** expression of SOX9 in GBM; **(D)** Western blot showing SOX9 protein expression in glioblastoma (GBM) and adjacent normal brain tissues; **(E)** Quantification of SOX9 relative to GAPDH. Data are shown as mean ± SD from four independent experiments (N = 4). P < 0.05 by unpaired t-test. (*P < 0.05, **P < 0.01, ***P < 0.001, ****P < 0.0001).

SOX9 is highly expressed in many tissues in the human body, including the central nervous system (**Figure 1A**). RNA-seq data was downloaded in TCGA and GTEx formats processed uniformly through the toil process. By comparing the expression of SOX9 normal samples in TCGA/GTEx with the corresponding tumor samples in TCGA, SOX9 was found significantly high expressed in 14 types of cancer (**Figure 1B**), including GBM (**Figure 1C**). The Western blot experiment has confirmed that the expression of SOX9 is significantly higher in GBM compared to adjacent normal tissues (**Figure 1D**). Quantitative analysis confirmed that SOX9 protein levels were significantly elevated in GBM tissues compared to adjacent normal tissues (**Figure 1E**).

### Enrichment analysis of SOX9 and co-expressed genes in GBM patients

**Figure 2:**
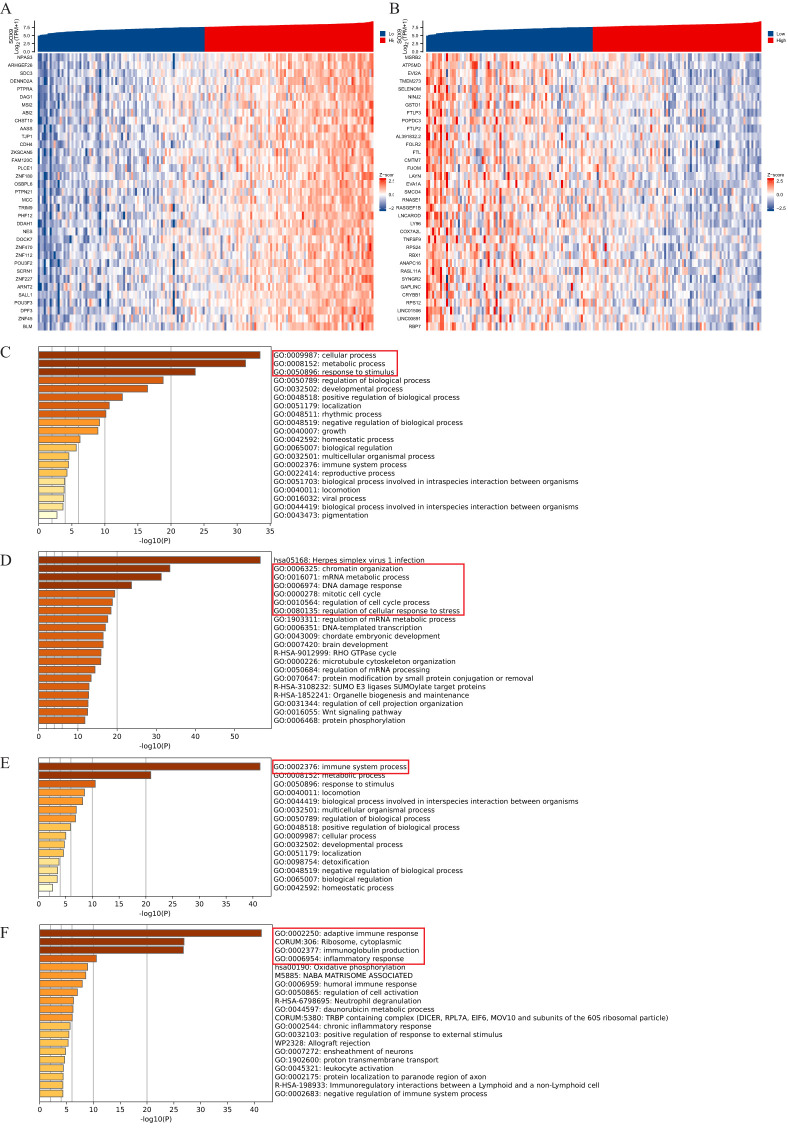
Enrichment analysis of SOX9 and co-expressed genes in GBM. **(A, B)** heatmaps of the top 35 positively/negatively related genes; **(C, D)** GO and KEGG pathway terms of positively related genes; **(E, F)** GO and KEGG pathway terms of negatively related genes.

Co-expressed genes of the SOX9 in GBM were obtained from the LinkedOmics database, and heatmaps of the top 35 positively/negatively related genes were drawn (**Figures 2A, B**). Then, we analyzed the GO and KEGG pathway terms of these genes using the Metascape database. The results of positively related gens showed significant enrichment of the biological processes (BP) terms “cellular process” and some processes related to “growth and development” (**Figure 2C**). The results showed significant enrichment of top 20 clusters terms “chromatin organization”, “mRNA metabolic process”, “DNA damage response”, “mitotic cell cycle”, “regulation of cell cycle process” and “regulation of cellular response to stress” (**Figure 2D**). The results of negatively related gens were shown in **Figures 2E, F**.

### Enrichment analysis of DEGs in THCA with low- and high-expressed SOX9

**Figure 3:**
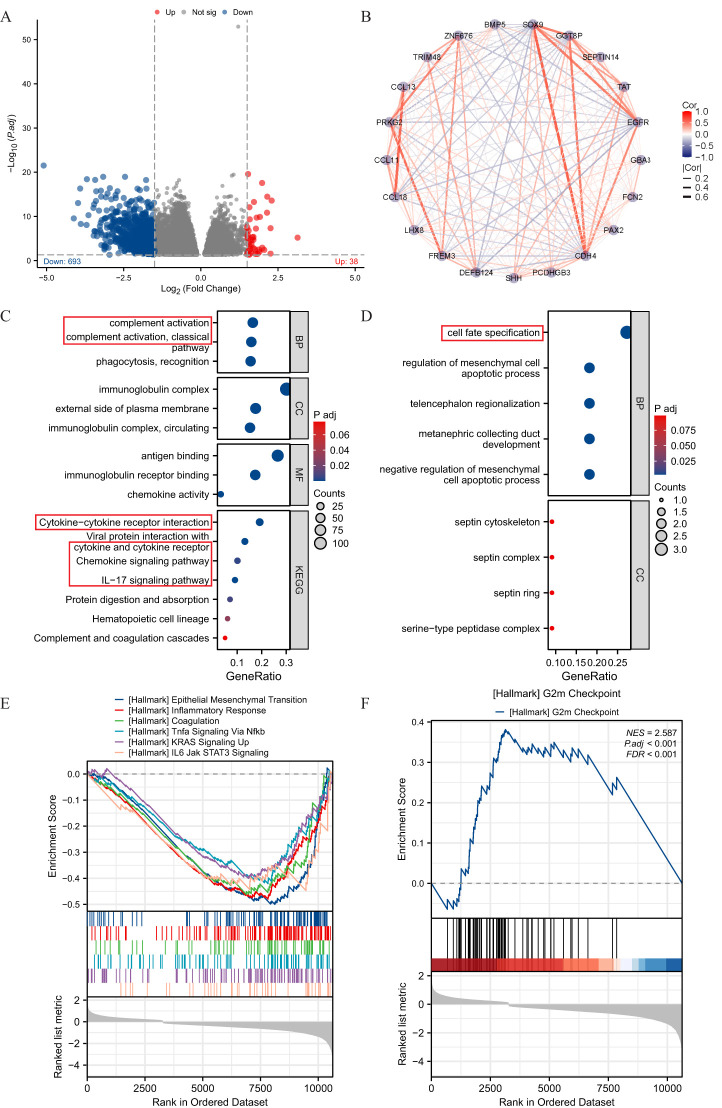
Enrichment analysis of DEGs in THCA with low- and high-expressed SOX9. **(A)** volcano plot of DEGs; **(B)** PPI network of SOX9-related DEGs; **(C, D)** GO and KEGG pathway terms of up- and down-regulated gens; **(E, F)** GSEA analysis between high- and low- SOX9 expression datasets.

The high- and low-expression groups’ gene expression profiles were analyzed for differences in the median mRNA expression. A total of 731 DEGs, including 38 upregulated and 693 down-regulated, were identified statistically significant between SOX9 high- and low-expressed groups (|log FC| >2 and adj P <0.05) (**Figure 3A**). The network of TREM1-related DEGs was constructed by STRING, with a threshold of 0.4. The PPI network was displayed by Cytoscape-MCODE (**Figure 3B**). The results of up-regulated gens showed significant enrichment of terms “cell fate specification” (**Figure 3C**). The results of down-regulated gens showed significant enrichment of terms “complement activation”, “cytokine−cytokine receptor interaction” and “cytokine receptor” et al. (**Figure 3D**). To further understand the biologic pathways involved in GBM with different SOX9 expression levels, GSEA was performed between low- and high-SOX9 expression datasets to identify critical signaling pathways. Significant differences (FDR <0.05, adj P <0.05) were observed in the enrichment of MSigDB Collection (C2.all.v7.0.symbols.gmt) of these pathways. G2/M Checkpoint was enriched in SOX9 high-expression phenotype (**Figure 3E**). In the low expression of SOX9 phenotypes, epithelial mesenchymal transition, inflammatory response, coagulation, TNF-α signaling via NF-κB, KRAS signaling up, and IL-6-JAK STAT3 signaling presented significantly enriched (**Figure 3F**).

### Association between SOX9 expression and clinical features of GBM

**Figure 4:**
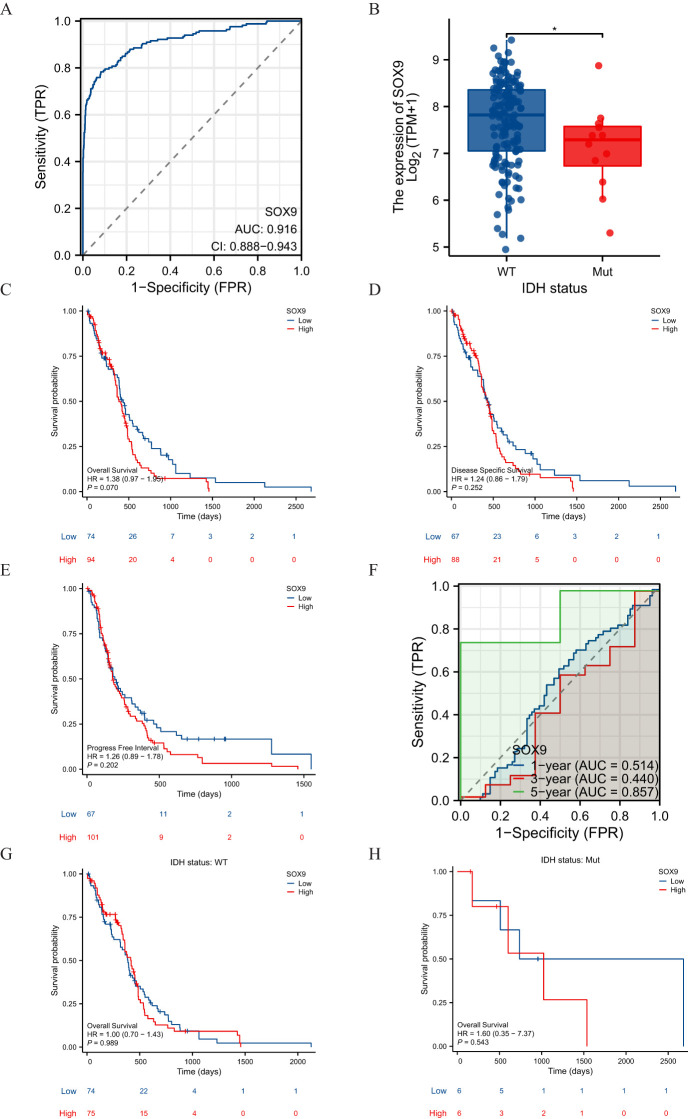
Diagnostic and prognostic value of SOX9 in GBM. **(A)** ROC curve showing the diagnostic accuracy of SOX9 in GBM. **(B)** Comparison of SOX9 expression between IDH-wildtype and mutant GBM (Wilcoxon test, P < 0.05). **(C–E)** Kaplan-Meier survival curves indicating that high SOX9 expression correlates with poorer prognosis. **(F–H)** Predictive performance of the model at 1-, 3-, and 5-year survival. These findings suggest that SOX9 may serve as a clinically relevant biomarker for diagnosis and prognosis in glioblastoma.

The main clinical characteristics of GBM in TCGA was shown in **Table 1**. In this study, a total of 168 cases (59 females and 109 males) were analyzed. The median SOX9 expression [log2(TPM+1)] was regarded as the cut-off value. Correlation analysis (**Table 2**) suggested that SOX9 expression was significantly correlated with IDH status (P <0.05). Logistic analysis was applied to further verify the relationship between GBM the factor of IDH status and the SOX9 high-low dichotomy. As a result, high expression of SOX9 showed a significant positive correlation with IDH status (odds ratio [OR], 0.168; P <0.05). Furthermore, the potential value of SOX9 in GBM patients was examined by ROC curve analysis, with the AUC of 0.916, revealing that SOX9 was a potential biomarker (**Figure 4A**). Besides, the Wilcoxon Rank SUM test was used to compare the expression of SOX9 in patients with different IDH status features. SOX9 expression was significantly higher in IDH-wildtype GBM compared to IDH-mutant samples (P < 0.05, Wilcoxon rank-sum test), as shown in **Figure 4B**. The relationship between SOX9 expression and prognosis was analyzed in GBM patients by using Kaplan-Meier. Patients with high expression of SOX9 had a worse prognosis than those with low SOX9 expression (over survival [OS], hazard ratio [HR]=1.38 (0.97 – 1.95), P = 0.070; disease specific survival [DSS], HR=1.24(0.86 -1.79), P=0.252, progress free interval [PFI], HR= 1.26 (0.89 − 1.78), P = 0.202) (**Figures 4C–E**). In order to further explore the accuracy of SOX9 in the evaluation of the prognosis of GBM patients, we conducted ROC curve analysis in both the training cohort and the validation cohort. As shown in **Figure 4F**, in the TCGA cohort, the area under the curve (AUC) values at 1, 3 and 5 years were 0.514, 0.440, and 0.857, respectively. In addition, the Kaplan-Meier analysis was used to generate survival curves for SOX9 expression in the IDH-mutant subtype. Patients with high expression of SOX9 had a worse prognosis than those with low SOX9 expression: IDH status: WT (OS, HR = 1.00 (0.70 − 1.43), P = 0.989), IDH status: Mut (OS, HR = 1.60 (0.35 − 7.37),P = 0.543) (**Figures 4G, H**).

**Table 1:** Baseline data.

Characteristics	Low expression of SOX9	High expression of SOX9	P value
n	84	84	
IDH status, n (%)			0.012
WT	68 (42.2%)	81 (50.3%)	
Mut	10 (6.2%)	2 (1.2%)	
Gender, n (%)			0.628
Female	31 (18.5%)	28 (16.7%)	
Male	53 (31.5%)	56 (33.3%)	
Race, n (%)			0.290
Asian	2 (1.2%)	3 (1.8%)	
Black or African American	8 (4.8%)	3 (1.8%)	
White	74 (44.6%)	76 (45.8%)	
Age, n (%)			0.165
<= 60	48 (28.6%)	39 (23.2%)	
> 60	36 (21.4%)	45 (26.8%)	
Karnofsky performance score, n (%)			0.257
< 80	14 (10.9%)	22 (17.2%)	
> 80	46 (35.9%)	46 (35.9%)	

**Table 2:** Univariate logistic regression.

Characteristics	Total (N)	OR (95% CI)	P value
IDH status (Mut vs. WT)	161	0.168 (0.036 - 0.793)	**0.024**
Gender (Male vs. Female)	168	1.170 (0.620 - 2.206)	0.628
Age (> 60 vs. <= 60)	168	1.538 (0.837 - 2.828)	0.165
Karnofsky performance score (> 80 vs. < 80)	128	0.636 (0.290 - 1.395)	0.259

Bold values indicate statistical significance (P < 0.05).

### Prognostic model of SOX9 in GBM

**Figure 5:**
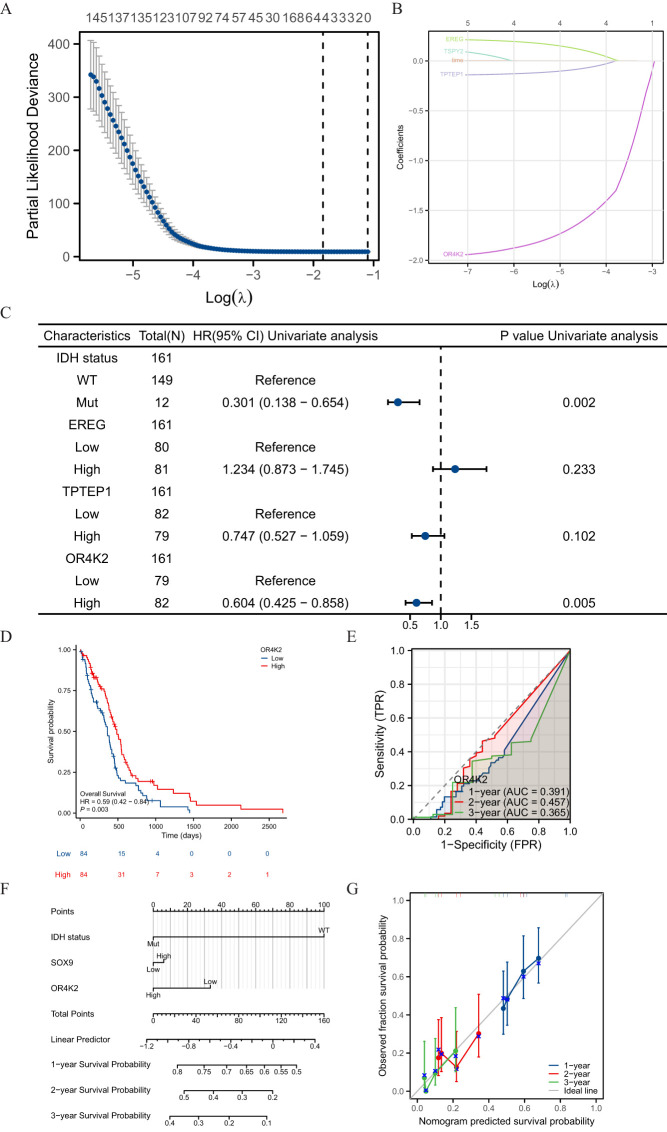
Prognostic model of SOX9 in GBM. **(A)** four genes screened by LASSO coefficients; **(B)** LASSO variable trajectories; **(C)** Forest plot presenting the results of COX regressions for four genes; **(D)** ROC curve over time with OR4K2 expression; **(E)** Kaplan-Meier curves in all GBM patients with OR4K2 expression; **(F)** Nomogram for predicting the probability of 1-, 3-, 5-year OS for GBM. **(G)** Calibration plot of the nomogram for predicting the probability of OS at 1, 3, and 5 years.

Screening was performed by LASSO coefficients to select non-zero variables that satisfied the coefficients of lambda. min, and 4 genes related with SOX9 were screened out. The risk score is calculated as follows: risk score = (0.09*EREG expression level - 0.027*TPTEP1 expression level - 0.309*OR4K2 expression level - 0.329*TSPY2 expression level) (**Figures 5A–B**). Multifactorial Cox regression analysis of these genes was performed using the survival R package and presented as forest plots (**Figure 5C**). Kaplan-Meier curves showed that OR4K2 high expression is a positive prognostic indicator (**Figure 5D**). The predictive power of the OS risk score assessed by the ROC curve over time was 0.391 AUC at 1 year, 0.457 at 3 years and 0.365 at 5 years with OR4K2 high expression (**Figure 5E**). A nomogram was constructed based on the Cox regression analysis results using the RMS R package (**Figure 5F**). IDH status, SOX9 expression and OR4K2 expression were included in the model. The points of each variable were accumulated and recorded as the total points. The probability of GBM patient survival at 1-, 3-, and 5-year was determined by drawing a line from the total point axis straight down to the outcome axis. The 1-year survival probability was determined by drawing a vertical line downward on the total point axis along the 140-direction ending axis, suggesting the probability of 1-year survival < 50%, both of the probability of 3- and 5-year < 5%. The prediction results of the nomogram calibration curve of OS were consistent with all patients’ observation results (**Figure 5G**).

### Immune infiltration analysis in GBM

**Figure 6:**
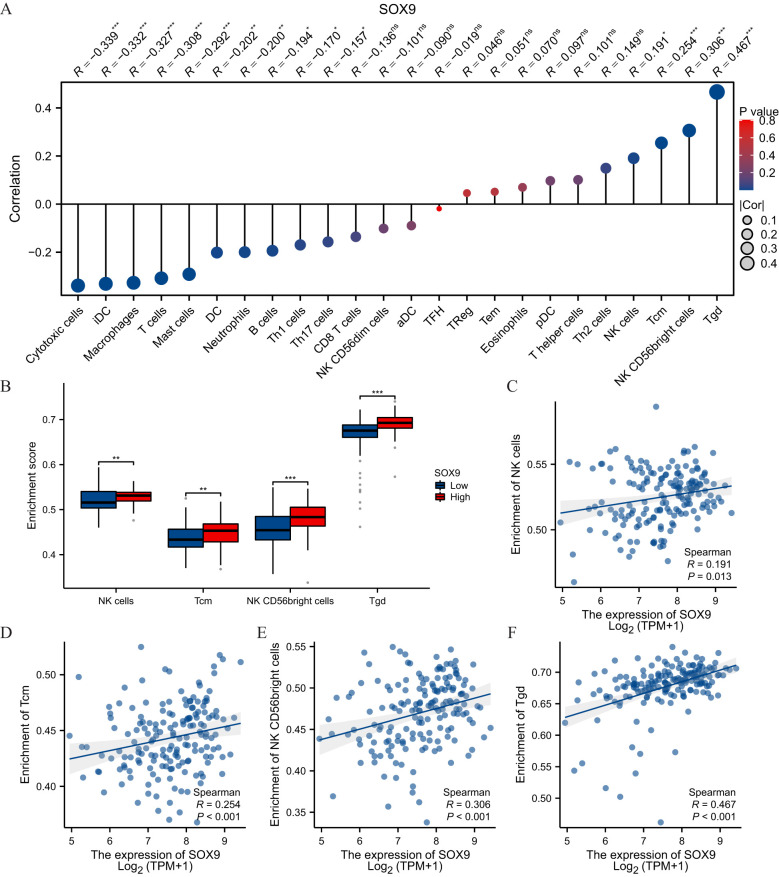
SOX9 was associated with immune infiltration in GBM. **(A)** The Lollipop chart showed a positive correlation between SOX9 and immune cells. The size of dots showed the absolute value of Spearman r. **(B)** Correlation between the relative enrichment score of NK cells, Tcm, NK CD56 bright, Tgd and the expression level of SOX9; **(C-F)** Infiltration of NK cells, Tcm, NK CD56 bright, Tgd high-SOX9 expressed. Statistical significance is indicated as follows: *P < 0.05; **P < 0.01; ***P < 0.001; ns, not significant.

Spearman correlation analysis showed that the expression level of TREM1 in the THCA microenvironment was correlated with the immune cell infiltration level quantified by SSGSEA. Specifically, SOX9 was positively associated with NK cells, Tcm, NK CD56bright and Tgd, but T cells inversely (**Figures 6A–F**).

### Relationship between SOX9 and immune checkpoints in GBM

**Figure 7:**
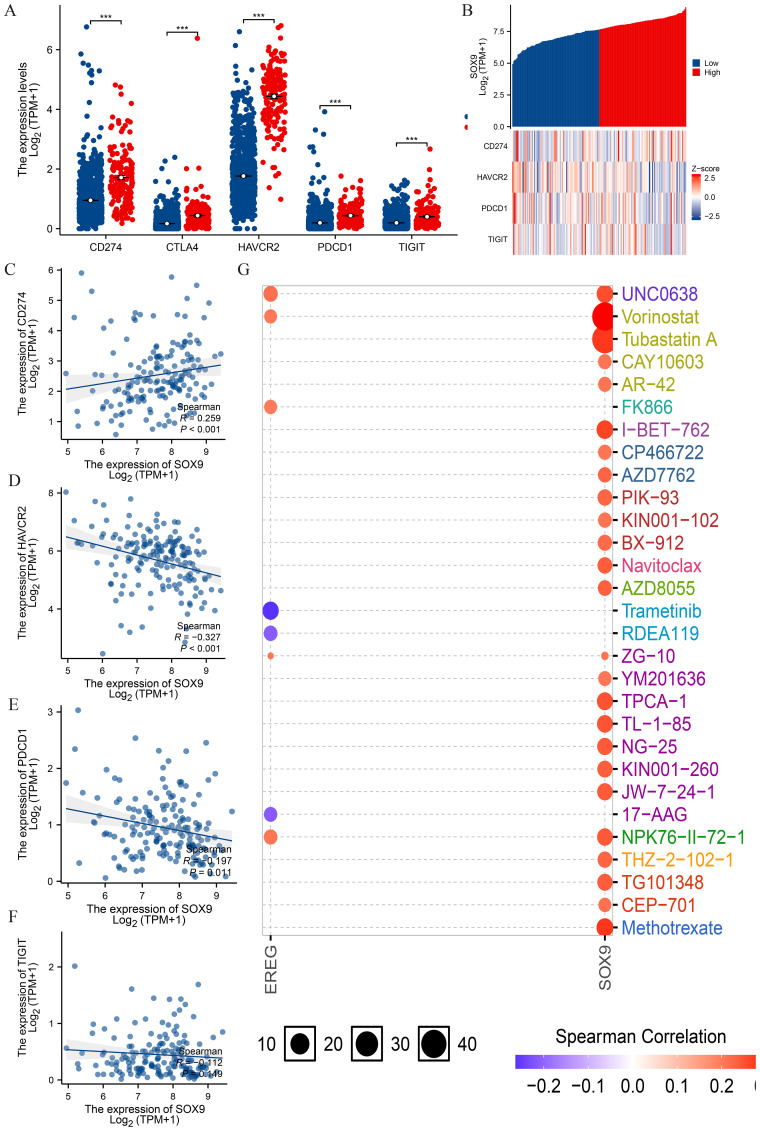
Correlation of SOX9 expression with immune checkpoints in GBM. **(A)** spearman correlation of SOX9 with expression of immune checkpoints in GBM; **(B)** co-expression heat map of SOX9 with expression of immune checkpoints in GBM; **(C-F)** Expression correlation between CD274, HAVCR2, PDCD1, TIGIT and SOX9; **(G)** SOX9 drug sensitivity analysis. Statistical significance is indicated as follows: ***P < 0.001.

PD1/PD-L1 and other immunization checkpoints are key immunization checkpoints that are responsible for tumor immune escape. Considering the potential oncogenic role of SOX9 in GBM, the relationship of SOX9 with PDCD1 and other immunization checkpoints was assessed. This correlation is visualized in the overall expression heatmaps and scatter plots (**Figures 7A, B**). The results showed that MSMO1 was negatively correlated with the expression of CD274 (P<0.05) (**Figure 7C**). The results showed that MSMO1 was negatively correlated with the expression of HAVCR2, PDCD1 and TIGIT (P <0.05) (**Figures 7D–F**). These results demonstrate that tumor immune escape might be involved in SOX9 mediated carcinogenesis of GBM. Finally, we used GSCALite online tool and TISDB database to analyze the relationship between the expression of SOX9 and sensitivity to current therapies. The result indicated that the expression levels of SOX9 were positively correlated with sensitivity to the most current cancer-targeted drugs or chemotherapy drugs (**Figure 7G**). Thus, SOX9 could represent a new target for predicting drug sensitivity and for developing multitarget combined therapy.

## Discussion

In recent years, there has been growing recognition of the carcinogenic role of SOX9, with experimental studies identifying its potential cancer-promoting effects in various malignant tumors, including gastric cancer and lung adenocarcinoma (16, 25). However, the role and mechanism of SOX9 in the development of GBM remain poorly understood, and specific bioinformatics analyses are lacking.

This study utilized data mining and analysis from the TCGA and GTEx databases, revealing that SOX9 expression was significantly upregulated in multiple tumor tissues, including GBM, compared to adjacent normal tissues. ROC curve analysis demonstrated an AUC of 0.916 for SOX9, indicating its potential diagnostic value. Western blot experiments further validated the elevated expression of SOX9 in GBM patient tissues. GSEA showed that high SOX9 expression was closely associated with the “G2M” checkpoint in the cell cycle. The cell cycle is a critical regulator of cell proliferation, and its dysregulation is a hallmark of cancer (26). Regulation of cell cycle progression is a defining feature of malignant cells. Conversely, low SOX9 expression was associated with pathways related to inflammation, including “inflammatory response,” “TNF-α signaling via NF-κB,” “KRAS signaling up,” and “IL-6-JAK STAT3 signaling.” Collectively, these findings suggest that SOX9 plays a crucial role in the pathogenesis of GBM by influencing cancer-related pathways. Consequently, SOX9 may serve as a promising therapeutic target for GBM.

Notably, high SOX9 expression was associated with poor prognosis in a subgroup of GBM patients with SOX9 mutations. IDH mutations are common in human malignancies and are found in over 80% of WHO grade II/III gliomas (27). In WHO grade IV GBM, IDH mutations are frequently observed in secondary GBM, accounting for 73% of clinical cases. Lower-grade gliomas with IDH mutations often recur and undergo malignant transformation to higher grades (28). Our findings reveal that high SOX9 expression in GBM patients with IDH mutations correlates with poor prognosis. Further studies are required to confirm the effects of elevated SOX9 expression on IDH-mutant GBM and elucidate the underlying mechanisms. This result further supports the potential role of SOX9 in promoting the aggressive phenotype of IDH-wildtype GBM and highlights its utility as a subtype-specific prognostic indicator.

To enhance prognosis prediction, we constructed a nomogram by integrating SOX9 expression, IDH mutation status, and OR4K2. The calibration curve showed excellent agreement between the nomogram’s predicted 1-year, 3-year, and 5-year overall survival (OS) probabilities and the observed outcomes. This indicates that the prognostic model has robust predictive capabilities. From a clinical perspective, the model provides personalized prognostic scores for individual GBM patients, offering a valuable tool for tailored patient management.

Immune cell infiltration into tumors significantly influences treatment outcomes and the prognosis of cancer patients. In this study, immune infiltration analysis revealed that high SOX9 expression was positively correlated with increased CD56 (bright) NK cell infiltration. Natural killer (NK) cells, lymphocytes of the innate immune system, play a pivotal role in defending against viral infections and tumors. Human NK cells are classified into two major subsets: CD56 (bright) and CD56 (dim) (29). CD56 (bright) NK cells are considered precursors to CD56 (dim) cells and are relatively immature. Compared to CD56 (dim) NK cells, CD56 (bright) cells produce higher levels of cytokines but exhibit lower cytotoxicity (30–32).

Recent studies suggest that CD56 (bright) NK cells may promote tumor progression rather than prevent it. Their accumulation has been reported in the tumor microenvironments of breast, colorectal, and lung cancers, where they are associated with immune evasion, angiogenesis, and reduced cytolytic function (33–35). Moreover, the tumor cytokine milieu may skew CD56 (bright) NK cells towards a pro-tumorigenic phenotype, contributing to immune escape (11, 32). In light of our findings, the positive correlation between SOX9 expression and CD56 (bright) NK cell infiltration may reflect SOX9’s role in promoting an immunosuppressive environment in glioblastoma, a hypothesis that warrants further mechanistic investigation.

NK cells have a dual role in tumor development. Traditionally, they have been regarded as essential for immune surveillance, contributing to anti-tumor activity (36). However, recent studies suggest that CD56 (bright) NK cells may promote tumor progression (35, 37). For instance, increased CD56 (bright) NK cell infiltration has been observed in colorectal and breast cancers (33, 34). Mechanisms underlying this pro-tumor activity include promoting tumor angiogenesis, enabling immune escape, and losing the ability to target tumor stem cells (11, 38). Cytokines in the tumor microenvironment also regulate the tumor-promoting behavior of CD56 (bright) NK cells (39).

In this study, CD56 (bright) NK cell infiltration was positively correlated with SOX9 expression. However, the potential role of COMMD7 in regulating CD56 (bright) and CD56 (dim) NK cells, as well as their involvement in immune escape in GBM, warrants further investigation.

Effective immunotherapy requires both adequate immune cell infiltration into the tumor microenvironment and sufficient expression of immune checkpoints (40). Immunotherapy has emerged as a promising treatment for malignant tumors, with immune checkpoint inhibitors achieving remarkable success in recent years (41, 42). For example, inhibitors targeting PD-1 and PD-L1 have demonstrated significant efficacy in treating melanoma, non-small cell lung cancer, renal cancer, and other solid tumors (43, 44). Given the success of immune checkpoint blockade therapies, this study also examined the relationship between SOX9 and immune checkpoints. Results revealed that high SOX9 expression was strongly associated with CD274, HAVCR2, and PDCD1 in GBM. Drug sensitivity analysis further suggested that multiple drugs targeting SOX9 are available, indicating that targeting SOX9 could enhance the efficacy of GBM immunotherapy. These findings raise the possibility that inhibition of SOX9 could enhance the effectiveness of immune checkpoint inhibitor therapy in GBM. Given its association with immunosuppressive NK cell infiltration and key checkpoint molecules such as PDCD1 and HAVCR2, SOX9 may act as an upstream regulator of immune evasion mechanisms. Additionally, drug sensitivity profiling supports the hypothesis that tumors with high SOX9 expression might respond better to certain targeted agents, such as HDAC and PI3K/mTOR inhibitors, which are known to influence immune signaling. Recent studies have further shown that SOX9 suppresses antitumor immunity in KRAS-driven lung adenocarcinoma (16), is correlated with immune-suppressive pathways across cancers (15), and promotes an immunosuppressive microenvironment in gastric cancer (13). Thus, SOX9 represents a promising immunomodulatory target, and further experimental studies are warranted to evaluate its therapeutic potential in combination with checkpoint blockade.

This study comprehensively analyzed SOX9 expression, gene mutations, immune cell infiltration, immune checkpoint associations, and its prognostic role in GBM. Data from multiple public databases revealed significantly increased SOX9 expression in GBM. Furthermore, SOX9 is upregulated across various cancer types and is strongly linked to poorer overall survival in GBM.

This study comprehensively analyzed SOX9 expression, gene mutations, immune cell infiltration, immune checkpoint associations, and its prognostic role in GBM. Data from multiple public databases revealed significantly increased SOX9 expression in GBM. Furthermore, SOX9 is upregulated across various cancer types and is strongly linked to poorer overall survival in GBM.

## Conclusion

In conclusion, this study highlights the critical role of SOX9 in GBM. SOX9 expression, gene mutations, immune cell infiltration, and immune checkpoint associations were systematically analyzed. Findings revealed that SOX9 is significantly upregulated in GBM and other cancer types, and its high expression is strongly associated with worse overall survival in GBM. These results suggest that targeting SOX9 holds potential as a therapeutic strategy to improve GBM outcomes.

## Data Availability

All data used in this study were obtained from publicly available databases unless otherwise stated. RNA-seq data and clinical information were downloaded from The Cancer Genome Atlas (TCGA) and Genotype-Tissue Expression (GTEx) databases. Co-expression data were retrieved from the LinkedOmics platform. Drug sensitivity and immune infiltration data were obtained from GSCALite and TISIDB. Protein expression information was retrieved from the Human Protein Atlas (HPA). Functional enrichment and network analyses were performed using Metascape and STRING databases. The Western blot experimental data generated by the authors from patient-derived glioma tissues are available upon reasonable request.
